# 口服美法仑作为多发性骨髓瘤自体造血干细胞移植预处理方案的疗效和安全性

**DOI:** 10.3760/cma.j.cn121090-20260126-00060

**Published:** 2026-07

**Authors:** 景 王, 琳 龚, 萌萌 潘, 诗炜 金, 坚青 糜, 卫平 章, 捷 许

**Affiliations:** 1 上海交通大学医学院附属瑞金医院血液内科，上海 200025 Department of Hematology, Ruijin Hospital Affiliated to Shanghai Jiao Tong University School of Medicine, Shanghai 200025, China; 2 蚌埠市第三人民医院血液内科，蚌埠 233000 Department of Hematology, The Third People's Hospital of Bengbu, Bengbu 233000, China; 3 湘西土家族苗族自治州人民医院血液内科，吉首 416000 Department of Hematology, Xiangxi Autonomous Prefecture People's Hospital, Jishou 416000, China

**Keywords:** 多发性骨髓瘤, 美法仑, 口服, 自体造血干细胞移植, 生物利用度, Multiple myeloma, Melphalan, Autologous hematopoietic stem cell transplantation, Biological availability

## Abstract

**目的:**

探索口服美法仑作为多发性骨髓瘤（MM）自体造血干细胞移植（auto-HSCT）预处理方案的疗效和安全性。

**方法:**

本项回顾性队列研究收集2022年7月至2023年10月在上海交通大学医学院附属瑞金医院进行auto-HSCT的MM患者的临床资料。根据美法仑的应用方式分为口服组和静脉组，观察两组患者移植后粒细胞、血小板植入时间（ET），移植后疗效，长期预后及治疗期间出现的不良事件。采用质谱技术检测患者美法仑血药浓度。

**结果:**

共纳入48例新诊断MM患者，口服组12例，静脉组36例。造血干细胞回输后，口服组中位ANC降至最低值时间（TTLL）和ET均较静脉组延长［TTLL：9.0（7.0～13.0）d对7.0（5.0～12.0）d，*P*<0.001；ET：12.0（10.0～14.0）d对11.0（9.0～12.0）d，*P*<0.001］；血小板表现出相似的差异［TTLL：10.0（9.0～15.0）d对9.0（6.0～13.0）d，*P*＝0.004；ET：14.5（11.0～17.0）d对12.0（9.0～19.0）d，*P*＝0.002］。口服组中位ANC最低值较静脉组高［（0.11（0.01～0.24）×10^9^/L对0.02（0.01～0.15）×10^9^/L，*P*<0.001］，两组中位PLT最低值的差异无统计学意义（*P*>0.05）。口服组与静脉组粒细胞缺乏（<0.5×10^9^/L）和PLT降低（<20×10^9^/L）中位持续时间的差异均无统计学意义（*P*值均>0.05）。非血液学不良反应方面，口服组胃肠道不良反应的发生率较静脉组显著降低（恶心：50.0％对100.0％，*P*<0.001；呕吐：8.3％对69.5％，*P*＝0.001；腹泻：50.0％对83.3％，*P*＝0.030）。两组口腔黏膜炎、肝肾功能损害的发生率差异均无统计学意义（*P*值均>0.05）。两组移植后疗效获得非常好的部分缓解以上比例及完全缓解率的差异也均无统计学意义（*P*值均>0.05）。中位随访时间37.9（31.0～49.4）个月，口服组和静脉组中位无进展生存期分别为未达到和35.9个月（95％*CI*：28.3个月～未达到），差异无统计学意义（*P*>0.05），两组的中位总生存期均未达到。6例患者（口服组3例，静脉组3例）接受了美法仑药代动力学评价，两组达到血药峰浓度的时间均在化疗开始后30 min，口服组平均浓度-时间曲线下面积是静脉组的82.2％。

**结论:**

口服美法仑作为MM患者auto-HSCT的预处理方案生物利用度良好，不良反应发生率低，临床疗效良好。

## Abstract (en)

**Objective:**

To investigate the safety and efficacy of oral melphalan as a conditioning regimen for patients with multiple myeloma（MM）who underwent autologous hematopoietic stem cell transplantation（auto-HSCT）.

**Methods:**

This retrospective cohort study collected clinical data of patients with MM who underwent auto-HSCT at Ruijin Hospital Affiliated to Shanghai Jiao Tong University School of Medicine from July 2022 to October 2023. Patients were divided into oral and intravenous groups according to the administration route of melphalan. Engraftment time（ET）, overall responses, long-term outcomes, and melphalan-associated toxicities were assessed. The pharmacokinetics of melphalan were detected using a mass spectrometer.

**Results:**

This study included 48 patients with MM who met the inclusion and exclusion criteria. Of these patients, 12 received tablet melphalan and 36 had the injection formulation. Upon engraft infusion, time to the lowest level（TTLL）and ET of neutrophils in the oral group were significantly longer than those in the injection group［TTLL: median, 9.0（7.0–13.0）d *vs* 7.0（5.0–12.0）d，*P*<0.001; ET: median, 12.0（10.0–14.0）d *vs* 11.0（9.0–12.0）d, *P*<0.001］. Such a lag also happened to the TTLL and ET of platelets in the oral group compared with the injection group［TTLL: median 10.0（9.0–15.0）d *vs* 9.0（6.0–13.0）d, *P*＝0.004; ET: median, 14.5（11.0–17.0）d *vs* 12.0（9.0–19.0）d, *P*＝0.002］. The lowest level of neutrophils in the oral group was higher than that in the injection group（0.11×10^9^/L *vs* 0.02×10^9^/L, *P*<0.001）, whereas the lowest level of platelets in both groups had no statistical difference（*P*>0.05）. The median duration of neutropenia（<0.5×10^9^/L）was comparable between the two groups. Regarding non-hematological side effects, patients who took melphalan orally had a remarkably lower frequency of gastrointestinal toxicities as opposed to those given intravenously（nausea: 50.0％ *vs* 100.0％, *P*<0.001; vomiting: 8.3％ *vs* 69.5％, *P*＝0.001; diarrhea: 50.0％ *vs* 83.3％, *P*＝0.030）. The incidence of oral mucositis and liver or kidney impairments did not differ between the two groups. Post-auto-HSCT efficacy evaluation demonstrated no differences in the percentages of patients obtaining a very good partial response and better outcomes, as well as in the improvement rate to complete remission. With a median follow-up time of 37.9（31.0–49.4）months, the median progression-free survival（PFS）of the oral and injection groups was not reached and 35.9 months（95％ *CI*: 28.3 months-not reached）, respectively. Median overall survival（OS）was not reached for either. Neither PFS nor OS reached statistical significance between the two groups. To examine the drug bioavailability, melphalan pharmacokinetics were tested in 6 patients, comprising 3 from the oral group and 3 from the injection group. The result demonstrated that both formulations reached their peak levels in 30 min. Although the oral melphalan exposure on average was 82.2％ of the injection dosage form, there was no significant difference in bioavailability.

**Conclusion:**

Oral melphalan, as a conditioning regimen for auto-HSCT in paients with MM, demonstrated favorable bioavailability, a low incidence of adverse reactions, and good clinical efficacy.

自体造血干细胞移植（auto-HSCT）是适合移植的新诊断多发性骨髓瘤（MM）患者的一线治疗，大剂量美法仑（200 mg/m^2^）预处理方案因清髓效果佳、抗肿瘤能力强、免疫抑制充分，成为国内外指南推荐的标准预处理方案[1]–[2]。

临床上，美法仑静脉注射制剂是应用较为普遍的剂型，与传统的含丙二醇的静脉制剂（PG-Mel）相比，经环糊精技术改良的美法仑（PGF-Mel）具有更强的药物稳定性[3]，但其胃肠道不良反应、黏膜炎性反应、肾脏不良反应等依然显著[4]–[5]，增加了患者auto-HSCT过程中的感染、出血风险。美国梅奥诊所的大样本研究显示，与PG-Mel相比，PGF-Mel无法降低药物相关不良反应、改善移植后疗效[6]。黄文阳等[7]分析了125例接受大剂量PGF-Mel预处理后进行auto-HSCT的MM患者的非血液学不良反应，125例（100％）恶心，100例（80％）呕吐，125例（100％）发生口腔黏膜炎，125例（100％）腹泻，112例（89.6％）发生感染。这些不良反应的严重程度被认为与美法仑在体内的总暴露量有关[8]。

口服美法仑以小剂量形式被广泛应用于初诊MM患者的诱导和巩固治疗中[9]。而大剂量口服制剂用于auto-HSCT的研究较少。国外有一项研究比较了口服与静脉应用美法仑作为MM患者auto-HSCT预处理方案的远期疗效，显示两组患者的无进展生存（PFS）率和总生存（OS）率无显著差异[10]。国内近期研究显示[11]，大剂量口服美法仑在MM患者auto-HSCT期间未诱发黏膜炎，而静脉输注美法仑患者黏膜炎发生率为71.4％；口服和静脉组≥3级腹泻的发生率分别为13.8％和60.0％，提示口服美法仑的不良反应发生率较静脉组低，但该特点是否与药代动力学相关尚不清楚。

为明确大剂量口服美法仑在接受移植的中国MM患者中的长期疗效和安全性，及其在患者体内的生物利用度，本研究对auto-HSCT前接受口服美法仑（总剂量200 mg/m^2^）预处理的12例MM患者开展了回顾性分析，将同期接受静脉PGF-Mel的患者作为对照组，评估两组的疗效和不良反应，并检测口服组与静脉组的血药浓度，比较两组患者对美法仑的生物利用度。

## 病例与方法

1. 病例：本研究为回顾性队列研究，患者纳入标准：①2022年7月至2023年10月在上海交通大学医学院附属瑞金医院血液内科移植病区接受auto-HSCT的新诊断MM患者；②预处理方案采用口服或静脉输注美法仑；③美法仑预处理总剂量为200 mg/m^2^。

患者排除标准：①合并浆细胞白血病、淀粉样变性、POEMS综合征；②复发进展MM；③各种原因未接受移植后2个月疗效评估。

本研究经上海交通大学医学院附属瑞金医院伦理委员会审批（批件号：2024-550），所有患者均签署书面知情同意书。在排除基础疾病影响的前提下，对于能够接受大剂量美法仑预处理的患者，告知其美法仑的用法、用量、相关不良反应、不同剂型的用药方式和费用后，根据患者意愿选择剂型。

2. 移植前诱导治疗方案：所有纳入研究的患者均在诱导治疗后获得部分缓解（PR）以上的疗效并完成自体外周血造血干细胞的采集，CD34^+^细胞数≥2×10^6^/kg。

诱导治疗采用以硼替佐米为基础的方案，包括硼替佐米+阿霉素/环磷酰胺+地塞米松±来那度胺±达雷妥尤单抗、硼替佐米+地塞米松±来那度胺±达雷妥尤单抗。高危细胞遗传学异常（HRCA）包括t（4;14）、t（14;16）、t（14;20）、del（17p）。

3. 造血干细胞动员及采集：自体外周血造血干细胞的动员采用化疗动员方案（环磷酰胺+依托泊苷+粒细胞集落刺激因子）或稳态动员方案（粒细胞集落刺激因子+普乐沙福）。使用Spectra Optia血液分离仪（日本Terumo公司）采集造血干细胞，流式细胞术检测CD34阳性细胞的比例。采集过程中予葡萄糖酸钙注射液预防低钙血症，术后监测血钾，血钾低者补钾。采集造血干细胞后加入造血干细胞低温保护剂放入液氮冻存。

4. 移植物淋巴细胞流式细胞术检测：取10 µl新鲜外周血造血干细胞，与一组标记淋巴细胞的抗体混合，包括抗人CD45（QB500，北京旷博生物技术股份有限公司）、CD3（APC R700，美国BD Biosciences公司）、CD4（APC-Cy7，北京旷博生物技术股份有限公司）、CD8（APC，美国BioLegend公司）、CD16（FITC，美国Beckman公司）、CD56（FITC，美国BD Biosciences公司）和CD19（PE-Cy5，美国Beckman公司）。在上述混合物中添加PBS补充至100 µl，室温下孵育15 min。加入300 µl红细胞裂解缓冲液去除红细胞，孵育并离心后，在BD FACSLyric™流式细胞仪（美国BD Biosciences公司）上检测样本，并使用Kaluza V1.2软件分析数据。

5. 预处理方案及治疗期间出现的不良事件（TEAE）的防治：造血干细胞回输日记录为第0天（d0），在回输日前2天（d−2）或d−3～d−2接受预处理方案。

口服组在早餐后2 h开始口服美法仑片剂，2 h内摄入当日要求的剂量，且与午餐间隔2 h以上。服用美法仑片剂期间，除饮用水外，禁止服用食物和其他药品。3例接受血药浓度检测的患者，在d−2当日接受总剂量（200 mg/m^2^），其余9例患者2 d完成美法仑治疗，每日口服100 mg/m^2^。

静脉组患者接受PGF-Mel。3例接受美法仑药物浓度检测的患者在d−2当日接受总剂量（200 mg/m^2^），其余33例患者2 d完成美法仑治疗，每日剂量为100 mg/m^2^。

两组患者在预处理化疗期间均接受冰盐水、复方氯己定含漱液、碳酸氢钠和氯化钠混合液漱口保护口腔黏膜。采用福沙匹坦双葡甲胺（d−3～d1）、昂丹司琼（d−3～d10）预防性止吐。自体造血干细胞回输后，中性粒细胞绝对计数（ANC）<1×10^9^/L时予粒细胞集落刺激因子，予抗生素预防感染，输注人免疫球蛋白增强免疫力。PLT<30×10^9^/L时输注去除白细胞的单采血小板。

6. 美法仑血药浓度测定：口服组和静脉组各3例患者测定美法仑血药浓度，采样标本为血清，每例患者在用药前，用药后30 min、1 h、2 h、3 h、6 h、24 h共7个时间点接受血清取样。样本经高速离心机离心（700×*g*）后，提取黄色清亮的上清液用于检测。美法仑的标准品（TRC-M216903）购自上海优宁维生物科技股份有限公司。血药浓度检测在上海药物代谢研究中心，采用质谱技术完成（SCIEX Triple Quad™ 6500^+^型三重四极杆串联质谱仪，序列号CF23971712，上海爱博才思分析仪器贸易有限公司），并利用Analyst 1.6.3软件（美国SCIEX公司）进行数据分析。

7. 疗效和TEAE评估：auto-HSCT后2个月评估疗效，疗效评估采用国际骨髓瘤工作组（IMWG）疗效标准[12]，包括严格意义的完全缓解（sCR）、完全缓解（CR）、非常好的部分缓解（VGPR）、PR。根据美国国家癌症研究所不良事件常用术语标准（CTCAE）5.0版评估不良反应，分级为1级（轻度）、2级（中度）、3级（重度）、4级（危及生命）、5级（死亡）。根据世界卫生组织（WHO）口腔毒性反应量表评估口腔黏膜炎，分级为0级、1级、2级、3级、4级、5级。

8. 随访：通过查阅电子病历、电话等方式随访。随访截止日期为2025年9月5日。中位随访时间为37.9（31.0～49.4）个月。

9. 统计学处理：应用SPSS 24.0和GraphPad Prism 8.0.2软件进行数据分析。计量资料以中位数（范围）表示，计数资料以例数（百分比）表示。分类变量采用*χ*^2^检验（或Fisher's精确检验）。连续变量先进行正态性检验，符合正态分布采用Student's *t*检验进行组间比较，不符合正态分布采用Mann-Whitney *U*检验。生存分析采用Kaplan-Meier法，使用R语言*Z*检验比较组间生存率。*P*<0.05为差异有统计学意义。

## 结果

1. 临床特征：共纳入48例MM患者，口服组12例，静脉组36例。口服组中位年龄59.5（48.0～65.0）岁，男性6例（50.0％）；IgG型4例（33.3％），IgA型5例（41.7％），IgD型1例（8.3％），轻链型1例（8.3％），不分泌型1例（8.3％）；DS分期：Ⅰ～Ⅱ期3例（25.0％），Ⅲ期9例（75.0％）；5例（41.7％）患者携带HRCA。静脉组中位年龄55.5（37.0～69.0）岁，男性21例（58.3％）；IgG型15例（41.7％），IgA型10例（27.8％），IgD型3例（8.3％），轻链型7例（19.4％），不分泌型1例（2.8％）；DS分期：Ⅰ～Ⅱ期4例（11.1％），Ⅲ期32例（88.9％）；9例（25.0％）患者携带HRCA。

口服组和静脉组中位诊断至移植时间分别为8（6～10）个月和9（4～15）个月。口服组分别有8例（66.7％）和4例（33.3％）患者接受造血干细胞化疗动员和稳态动员；静脉组分别有26例（72.2％）和10例（27.8％）患者接受造血干细胞化疗动员和稳态动员。回输CD34^+^细胞数方面，口服组和静脉组中位CD34⁺细胞输注量分别为3.6（1.8～13.8）×10^6^/kg和4.5（1.8～11.4）×10^6^/kg。移植物淋巴细胞成分方面，口服组CD4^+^T细胞（CD3^+^CD4^+^）、CD8^+^T细胞（CD3^+^CD8^+^）、NK细胞（CD3^−^CD16^+^CD56^+^）、B细胞（CD19^+^）占总淋巴细胞的比例分别为48.0％、30.0％、4.3％、3.9％，静脉组中上述细胞所占比例分别为43.9％、34.8％、6.2％、3.8％。

所有患者在诱导治疗阶段均接受了硼替佐米。口服组和静脉组分别有7例（58.3％）和23例（63.9％）接受来那度胺，7例（58.3％）和14例（38.9％）接受达雷妥尤单抗。移植前疗效方面，口服组1例（8.3％）PR，6例（50.0％）VGPR，4例（33.3％）CR，1例（8.3％）sCR；静脉组3例（8.3％）PR，8例（22.2％）VGPR，12例（33.3％）CR，13例（36.1％）sCR。

口服组和静脉组上述基线特征的差异均无统计学意义（*P*值均>0.05）。

2. 给药方式对造血重建的影响：所有患者经历了粒细胞缺乏期（ANC<0.5×10^9^/L），口服组和静脉组中位ANC最低值分别为0.11（0.01～0.24）×10^9^/L和0.02（0.01～0.15）×10^9^/L，差异有统计学意义（*P*<0.001）。口服组和静脉组中位PLT最低值分别为6.0（1.0～35.0）×10^9^/L和6.0（1.0～27.0）×10^9^/L，差异无统计学意义（*P*＝0.977），口服组和静脉组各有2例患者的PLT未降低至20×10^9^/L以下。

口服组和静脉组中位ANC降至最低值时间（time to the lowest level，TTLL）分别为9.0（7.0～13.0）d和7.0（5.0～12.0）d（*P*<0.001），中位PLT的TTLL分别为10.0（9.0～15.0）d和9.0（6.0～13.0）d（*P*＝0.003）。

口服组和静脉组在auto-HSCT后均获得造血重建，两组的中位粒细胞植入时间（engraftment time，ET）分别为12.0（10.0～14.0）d和11.0（9.0～12.0）d（*P*<0.001），中位血小板ET分别为14.5（11.0～17.0）d和12.0（9.0～19.0）d（*P*＝0.002）。

口服组中位粒细胞缺乏持续时间为6.0（3.0～10.0）d，静脉组中位粒细胞缺乏持续时间为7.0（4.0～9.0）d，差异无统计学意义（*P*＝0.194）。口服组和静脉组PLT减少（<20×10^9^/L）的中位持续时间分别为5.0（0～10.0）d和4.0（0～8.0）d，差异无统计学意义（*P*＝0.313）。

3. TEAE：口服组和静脉组的血液学不良反应（ANC<0.5×10^9^/L）均为4级，达到清髓目标。两组的非血液学不良反应主要表现为消化道症状，其次为肝肾功能损害和感染，具体见表1。口服组的安全性优于静脉组，尤其减轻了胃肠道不良反应（表1）。

**表1:** 美法仑口服组和静脉组多发性骨髓瘤患者的非血液学不良反应比较［例（％）］

不良反应	口服组（12例）	静脉组（36例）	*χ*^2^值	*P*值
口腔黏膜炎			4.59	0.101
无	6（50.0）	7（19.4）		
1～2级	6（50.0）	27（75.0）		
3～4级	0（0）	2（5.6）		
恶心			20.68	<0.001
无	6（50.0）	0（0）		
1～2级	6（50.0）	35（97.2）		
3～4级	0（0）	1（2.8）		
呕吐			13.55	0.001
无	11（91.7）	11（30.6）		
1～2级	1（8.3）	24（66.7）		
3～4级	0（0）	1（2.8）		
腹泻			7.00	0.030
无	6（50.0）	6（16.7）		
1～2级	5（41.7）	15（41.7）		
3～4级	1（8.3）	15（41.7）		
肝功能损害			0.76	0.683
无	7（58.3）	24（66.7）		
1～2级	5（41.7）	11（30.6）		
3～4级	0（0）	1（2.8）		
肾功能损害			0.70	0.404
无	11（91.7）	35（97.2）		
1～2级	1（8.3）	1（2.8）		
感染			1.52	0.218
无	11（91.7）	27（75.0）		
有	1（8.3）	9（25.0）		

4. 移植后疗效：所有患者均未发生auto-HSCT相关死亡事件，在auto-HSCT后2个月接受移植后首次疾病评估。结果显示，两组整体治疗反应率（疗效达到PR及以上）均为100％。口服组1例（8.3％）PR，3例（25.0％）VGPR，8例（66.7％）CR/sCR，疗效达VGPR以上发生率较移植前未升高，CR/sCR率较移植前升高25.0％；静脉组1例（2.8％）PR，4例（11.1％）VGPR，31例（86.1％）CR/sCR，疗效达VGPR以上发生率较移植前升高5.6％，CR/sCR率较移植前升高16.7％（图1）。口服组与静脉组移植后2个月的疗效改善差异无统计学意义（*P*＝0.404）。

**图1:**
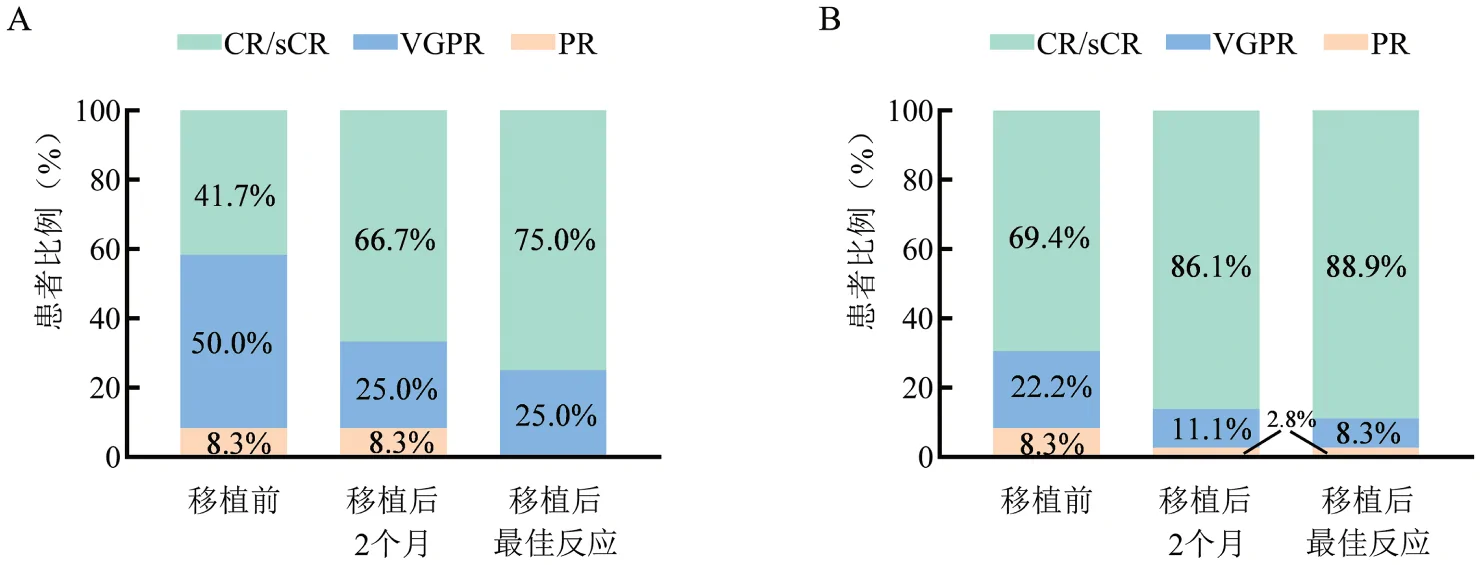
美法仑口服组（A）与静脉组（B）多发性骨髓瘤患者移植前后的疗效 **注** CR：完全缓解；sCR：严格意义的完全缓解；VGPR：非常好的部分缓解；PR：部分缓解

截至2025年9月5日，口服组最佳疗效：3例（25.0％）VGPR，9例（75.0％）CR/sCR；静脉组最佳疗效：1例（2.8％）PR，3例（8.3％）VGPR，32例（88.9％）CR/sCR（图1）。与移植前状态相比，口服组最佳疗效中1例（8.3％）从PR到VGPR，4例（33.3％）从VGPR到CR/sCR；静脉组最佳疗效中2例（5.6％）从PR到CR/sCR，5例（13.9％）从VGPR到CR/sCR。将缓解深度改善定义为疗效从PR/VGPR转变为CR/sCR，口服组与静脉组缓解深度改善率的差异无统计学意义（33.3％对19.4％，*P*＝0.322）。

5. 长期生存：口服组的中位PFS期未达到，静脉组的中位PFS期为35.9个月（95％*CI*：28.3个月～未达到），两组PFS的差异无统计学意义（*P*＝0.615）（图2A）。两组6个月PFS率均为100％。口服组与静脉组的12个月PFS率分别为83.3（95％*CI*：48.2～95.6）％和97.2（95％*CI*：81.9～99.6）％，差异无统计学意义（*P*＝0.211）。口服组与静脉组的36个月PFS率分别为58.3（95％*CI*：27.0～80.1）％和35.9（95％*CI*：8.5～65.3）％，差异无统计学意义（*P*＝0.303）。口服组和静脉组的中位OS期均未达到，差异无统计学意义（*P*＝0.354）（图2B）。

**图2:**
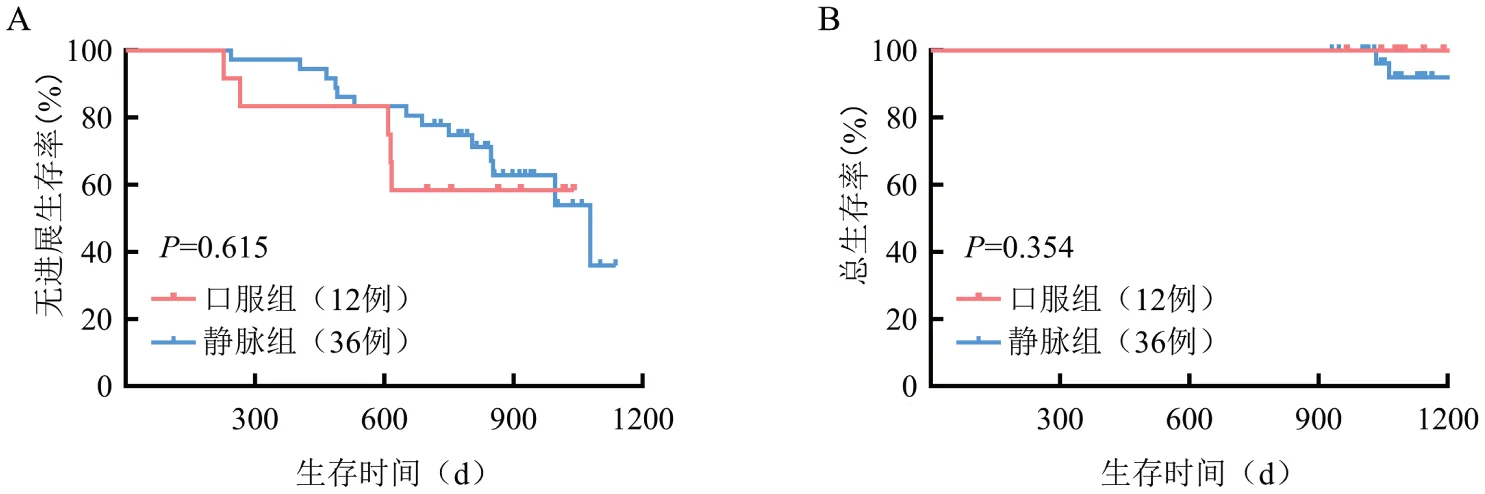
美法仑口服组与静脉组多发性骨髓瘤患者的无进展生存（A）与总生存（B）曲线

6. 血药浓度：为明确口服美法仑的生物利用度及其与静脉制剂的差异，6例患者（口服组3例，静脉组3例）监测了美法仑的血药浓度变化。6例患者均在d−2接受了美法仑总剂量，在用药前、后共7个时间点采集其血清样本并测定美法仑浓度。结果显示，口服组和静脉组的美法仑浓度均在给药后0.5 h达到高峰，后逐渐下降，24 h后低于检测下限。其中，口服组和静脉组的平均浓度峰值（C_max_）分别为（3 310±1 370）ng/ml和（4 010±1 640）ng/ml（**扫描本文首页二维码查阅附图1**）。口服组和静脉组的平均浓度−时间曲线下面积（AUC_0−∞_）分别为（7.12±2.85）µg·h^−1^·ml^−1^和（8.66±3.28）µg·h^−1^·ml^−1^，口服组的生物利用度是静脉组的82.2％（**扫描本文首页二维码查阅附图1**）。

## 讨论

MM是浆细胞恶性肿瘤，至今无法治愈。目前认为，遗传学异常及克隆演变是MM复发耐药的根本原因[13]，增加缓解深度、延长无病缓解期是MM的治疗目标。近20年来，auto-HSCT的普及在改善MM整体预后方面作出了重要贡献[14]。最新的全球血液和骨髓移植组统计数据显示，接受auto-HSCT的MM患者的中位PFS期可超过3年，中位OS期达到7.5年，较10年前升高了2倍[15]。

大剂量美法仑是MM行auto-HSCT的核心预处理方案，国内外指南建议以200 mg/m^2^作为标准使用剂量，但未对美法仑的剂型作出明确推荐。当前应用较多的美法仑剂型是PGF-Mel，其非血液学不良反应相当突出。EVOMELA研究表明，接受PGF-Mel预处理的患者均发生了非血液学不良反应，90％以上为消化道功能紊乱，包括腹泻、恶心、呕吐等，3级不良反应发生率达69％，4级达10％，包括败血症、便血[16]。尽管本研究应用了完善的止吐预防措施，但接受PGF-Mel的患者发生消化道不良反应的风险与国内外报道数据一致[6]–[7],[16]，提示临床常用的止吐药物无法有效控制美法仑静脉制剂诱发的恶心、呕吐反应。在预处理方案中采用口服美法仑可显著降低消化道症状的发生率及其严重程度，可能与人体吸收总量减少有关[17]。有报道指出，食物可降低美法仑的吸收总量[18]。鉴于此，本研究口服组患者均在胃排空状态下服药，以避免食物对美法仑吸收的影响。尽管如此，胃酸、高剂量美法仑对胃肠黏膜的毒性也可能阻碍吸收[19]，降低口服制剂的平均吸收量[17]。

本研究比较了口服与静脉接受美法仑患者的血药浓度。两种剂型美法仑达到C_max_的时间一致，提示口服制剂吸收迅速，然而口服组的C_max_和AUC_0−∞_为静脉组的80％左右，由于纳入的检测样本量较少，上述对比尚需在大样本研究中进一步验证。对照临床数据，口服美法仑在清髓时间上明显迟于静脉美法仑，尽管其骨髓抑制持续时间与静脉相似，但对粒细胞的抑制程度弱于静脉制剂，这很可能与口服制剂的生物暴露量略低有关。无论是静脉还是口服制剂，个体的AUC_0−∞_具有一定差异，可能与个体的肌酐清除率及遗传背景有关，如GSTP1、SLC7A5基因的多态性[20]–[21]。结合本研究，虽然患者基线肾功能均正常，但有2例（口服和静脉各1例）患者在接受美法仑后出现肌酐升高，2例患者均接受了单次美法仑给药方案和药代动力学研究，其C_max_和AUC_0−∞_较其他接受血药浓度检测的患者明显升高，并出现一过性肌酐清除率降低的情况，这可能与2例患者肾脏清除药物的速度较慢有关。一项针对大剂量静脉美法仑的药效学分析报告指出，高美法仑暴露量虽然是造成不良反应的危险因素，但可以显著延长MM患者的OS期[22]。通过体重和肌酐清除率预测美法仑的药代动力学和潜在的药物暴露量可以优化个体使用剂量，在达到药物最大疗效的同时，控制美法仑相关的移植不良反应[23]。

口服组疗效从PR+VGPR提升至CR/sCR的患者比例略高于静脉组，虽然差异无统计学意义，但可能与口服组移植前深度缓解的患者比例低有关。早期深度缓解被认为对PFS有重要的影响[24]。本研究显示，静脉组的1年PFS率略优于口服组。但两组的3年PFS率相似，提示远期疗效相仿。

PGF-Mel价格昂贵，是一部分患者放弃auto-HSCT的原因之一。本研究纳入的口服组患者均符合标准静脉制剂清髓条件，但因经济条件有限而选择口服片剂。因此，大剂量口服美法仑的临床应用为更多MM患者提供了接受auto-HSCT治疗的机会。

综上，口服美法仑能够显著降低严重不良反应的发生率，基本取得与静脉美法仑相当的临床疗效。但本研究纳入的例数较少，进行血药浓度检测的样本量有限，为进一步验证美法仑片剂的耐受性、有效性及其药代动力学，本研究组正在开展扩大病例的前瞻性临床研究（中国临床试验注册中心注册号ChiCTR2500097863），以优化口服美法仑在auto-HSCT中的临床应用剂量。

## Supplementary Material


